# [Corrigendum] Roles of the H19/microRNA‑675 axis in the proliferation and epithelial‑mesenchymal transition of human cutaneous squamous cell carcinoma cells

**DOI:** 10.3892/or.2023.8586

**Published:** 2023-06-14

**Authors:** Wenqing Zhang, Kaili Zhou, Xue Zhang, Chenglong Wu, Dan Deng, Zhirong Yao

Oncol Rep 45: 39, 2021; DOI: 10.3892/or.2021.7990

Following the publication of the above paper, a concerned reader drew to the authors’ attention that, in Fig. 4C on p. 8, the ‘Invasion, miR-675-inhibitor’ data panel appeared to contain an overlapping section with the ‘Invasion, mi-R675-inhibitor + pcDNA3.1-H19’ data panel for the SCL1 cell line, such that the data were likely to have been derived from the same original source, even though they were intended to show the results from differently performed experiments. After having examined the original data, the authors also realized that the ‘Inhibitor-NC’ and ‘miR-675-inhibitor’ data panels showing the migration assay experiments for the A431 cell line in the same figure part had also inadvertently been derived from the same original source.

After having been granted permission from the Editor of *Oncology Reports* to repeat the experiments shown in Fig. 4C, the revised version of Fig. 4, incorporating the new data for Fig. 4C, is shown on the next page. Note that these errors did not affect the overall conclusions reported in the study, and the repeated experiment yielded similar results to those obtained originally. The authors are grateful to the Editor for allowing them the opportunity to publish this corrigendum, and all the authors agree with the publication of this; furthermore, they apologize for any inconvenience caused to the readership of the Journal.

## Figures and Tables

**Figure 4.**
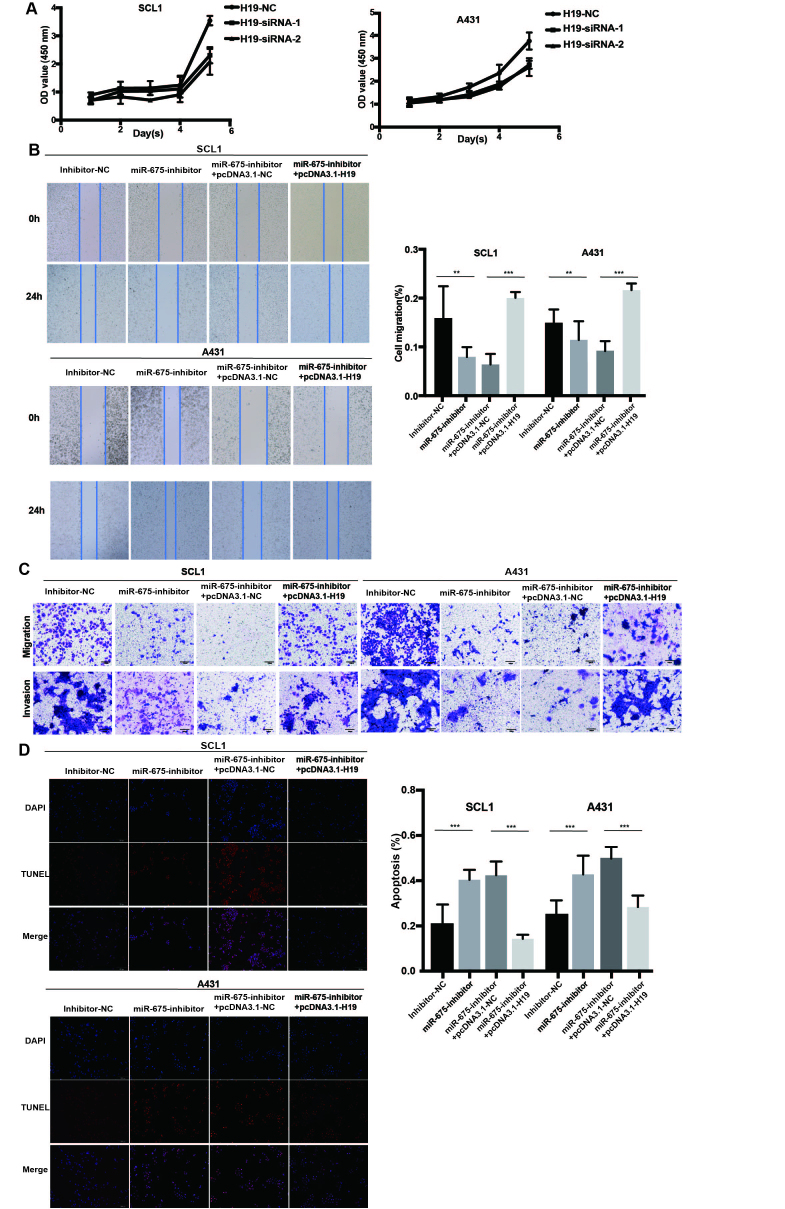
H19 and miR-675 knockdown inhibits cSCC cell proliferation, migration and invasion, and promotes apoptosis. (A) Proliferation of SCL1 and A431 cells transfected with H19-siRNAs was detected using a Cell Counting Kit-8 assay. (B) A wound healing assay was used to analyze the migration of cSCC cells transfected with inhibitor-NC, miR-675 inhibitor, miR-675-inhibitor + pcDNA3.1-NC and miR-675 inhibitor + pcDNA3.1-H19. Magnification, ×200. (C) Migration and invasion of cSCC cells transfected with inhibitor-NC, miR-675 inhibitor, miR-675-inhibitor + pcDNA3.1-NC and miR-675 inhibitor + pcDNA3.1-H19 were analyzed using Transwell assays. Magnification, ×200. (D) TUNEL assays were used to determine apoptosis in cSCC cells transfected with inhibitor-NC, miR-675 inhibitor, miR-675-inhibitor + pcDNA3.1-NC and miR-675 inhibitor + pcDNA3.1-H19. Magnification, ×200. **P<0.01 and ***P<0.001. miR, microRNA; siRNA, small interfering RNA; NC, negative control; cSCC, cutaneous squamous cell carcinoma.

